# Shake it off: exploring drivers and outcomes of autotomy in marine invertebrates

**DOI:** 10.1098/rsbl.2024.0015

**Published:** 2024-05-29

**Authors:** Sara Jobson, Jean-François Hamel, Annie Mercier

**Affiliations:** ^1^ Department of Ocean Sciences, Memorial University, St John’s (Newfoundland and Labrador), Canada; ^2^ Society for the Exploration and Valuing of the Environment, St Philips (Newfoundland and Labrador), Canada

**Keywords:** autotomy, breakage plane, voluntary limb loss, Echinodermata, Arthropoda, amputation

## Abstract

Autotomy refers to self-amputation where the loss of a limb or organ is generally said to be (1) in response to stressful external stimuli; (2) voluntary and nervously mediated; (3) supported by adaptive features that increase efficiency and simultaneously mediate the cost; and (4) morphologically delineated by a predictable breakage plane. It is estimated that this phenomenon has evolved independently nine different times across the animal kingdom, appearing in many different taxa, including vertebrate and invertebrate as well as aquatic and terrestrial animals. Marine invertebrates use this behaviour in a diversity of manners that have yet to be globally reviewed and critically examined. Here, published data from marine invertebrate taxa were used to explore instances of injury as an evolutionary driver of autotomy. Findings suggest that phyla (e.g. Echinodermata and Arthropoda) possibly experiencing high rates of injury (tissue damage or loss) are more likely to be able to perform autotomy. Additionally, this review looks at various morphological, physiological and environmental conditions that have either driven the evolution or maintained the behaviour of autotomy in marine invertebrates. Finally, the use of autotomic abilities in the development of more sustainable and less ecologically invasive fisheries is explored.

## 1. Introduction

Autotomy is broadly defined as the self-amputation of a limb or organ in response to an external stimulus. The theories explaining this phenomenon have shifted several times since it was coined by Leon Fredericq [1] from observations of limb loss in crustaceans. Since the 1800s, autotomy has received considerable attention, and its definition has evolved. Most authors now define autotomy based on loss of tissue/limb/organ that (1) occurs in response to stressful external stimuli; (2) is voluntary and nervously mediated; (3) is supported by adaptive features that increase efficiency and simultaneously mediate the cost; and whereby (4) these features delineate a predictable breakage plane [2–6]. On occasions, the definition of autotomy has been further expanded. Primarily, the term was co-opted by several other fields of biological science to describe animals that reproduce asexually through binary fission [7] or vertebrates that exhibit self-mutilation or denervation following neural degradation [8]. Because they fall outside the definition of autotomy presented above, these behaviours will not be covered in this review. Additionally, previous research has tightly coupled explanatory theories of regeneration and autotomy [9,10], As the former supports the overall benefit of the latter by mitigating how long an organism will experience increased vulnerability. While regeneration is an integral part of autotomy, it will not be covered in-depth within this review since the synthesis of relevant information on this topic has been completed by other authors [10–18].

Voluntary amputation is a puzzling behavioural phenomenon that appears across a wide variety of taxa, seemingly without a common evolutionary ancestor. Although it has been suggested for decades that autotomy had various evolutionary origins [19], taxonomically biased research made it difficult to support this claim until recently [3]. It is possible that research into the evolutionary origins of autotomy was also hindered by both the liberal use of the term autotomy and incorrect use of related terms (e.g. fission) across biological disciplines, making it difficult to track assessments of this behaviour within the available literature (for a list of search terms see electronic supplementary material, table S1). For example, fission implies sectioning of a body part for the purpose of asexual reproduction, however, it has been widely used to describe autotomy as well. While these processes often share physiological mechanisms, the drivers and outcomes are unique. To better explore limb loss outside of asexual reproduction, this review will focus solely on autotomy.

Nevertheless, autotomy is now being proposed to have evolved independently nine times [3]. The prolific demonstration of autotomy as a form of convergent evolution suggests strong and common driving forces behind its development [3]. This assumption is reinforced by the diversity of taxa that display autotomic abilities, although squamate reptiles have occupied the spotlight in this area of research since Aristotle first observed tail autotomy in lizards over 2000 years ago [2,15,20]. Research around vertebrate model species (all belonging to the same phylum) has informed much of our current understanding of the drivers and outcomes of autotomy. There is now a need to explore paradigms from a broader taxonomic perspective, considering species that exhibit a wide variety of body plans, life-history strategies and live in completely different environmental niches. Marine invertebrate taxa represent a large portion of animal diversity and thus provide many examples of autotomy (electronic supplementary material, table S2), offering an ideal framework to address knowledge gaps regarding the triggers and mechanisms involved in this adaptation.

**Figure 1.**
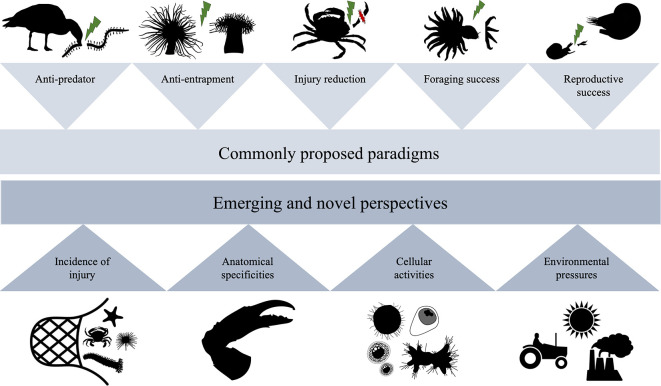
Commonly proposed paradigms and emerging and novel perspectives around autotomy in marine invertebrates. Green graphic (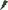) indicates auotomy and red graphic (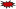) indicates a point of injury.

Globally, autotomy has been primarily described as a defence mechanism, whereby limbs or organs are discarded in response to the external stimuli associated with predator–prey interactions [10,21–24]. However, a resurgence of research on autotomy in recent years has outlined other situations where species employ this behaviour to increase their overall fitness (e.g. reproduction, anti-entrapment or injury reduction [25–27]). A crucial step in understanding autotomy in marine environments is characterizing common or emerging drivers (i.e. selective evolutionary pressures that increase the benefit of a specific behaviour; figure 1) and outcomes (i.e. the resulting costs and/or benefits of a specific behaviour appearing at organismal, population or ecological scales). This review explores autotomy in marine animals by outlining the commonly proposed paradigms and examining emerging concepts and new avenues for research. A final segment highlights how the autotomic abilities of selected marine species might have practical commercial applications.

## 2. Commonly proposed paradigms of autotomy

### (a) Anti-predation

The most common conclusion among researchers has been that autotomy is primarily an anti-predator behaviour [28]. This hypothesis suggests that the cost of autotomizing a limb and having to regrow it is acceptable in the face of a terminal predator attack. While this may seem obvious from a Darwinian perspective (i.e. increasing the chance an individual will be able to pass on its genes), ongoing research into post-autotomy recovery has refined our understanding of the cost versus benefit relationship. Not only are there immediate metabolic costs associated with healing or full regeneration of lost limbs [29–31], but there is also the difficulty or inability to forage, reproduce and defend against further predatory attacks during the regenerative period [32].

The selective pressure of predation has driven specialized and complementary features to appear in some autotomous species. For example, *Harmothoe imbricata* (Annelida: Polychaeta) presents luminescent elytra (scales) shown to draw predator focus to the part of the body that can autotomize [33]. Another example is the japanese sea lily *Metacrinus rotundus* (Echinodermata: Crinoidea) whose arms have the ability to continue moving post-shedding to distract the predator and provide additional escape time [34]. In the cosmopolitan sea slug genus, *Oxynoe* (Mollusca: Gastropoda) and the bivalve *Limaria hians* (Mollusca: Bivalvia), autotomy of the posterior margin and the tentacles, respectively, causes immediate excretion of noxious mucus with the purpose of deterring predators [35,36]. Each of these adaptations directly influence the efficiency of autotomy during predator–prey interactions and subsequently, support the hypothesis that predator pressures are an active and powerful driver of autotomy.

### (b) Anti-entrapment

While the threat of predation exerts strong selective pressure, the use of autotomy in non-predatory situations is not uncommon [37]. For example, the plumose sea anemone *Metridium senile* (Cnidaria: Anthozoa) is able to autotomize tentacles that have been entangled in conspecifics during intraspecific interaction [38]. Similarly, tentacle autotomy in *Aglantha digitale* (Cnidaria: Tachymedusae) has been documented when nematocyte discharge causes accidental entanglement in surrounding planktonic taxa [39]. In the burrowing filter feeder, *Lingula anatine* (Brachiopoda: Lingulata) coarse sediment inhibits locomotion, so autotomy of the pedicle is employed to support active burrowing [40].

Another example of anti-entrapment autotomy is escaping a failed moult where limbs are trapped. This has been studied in *Carcinus maenas* (Arthropoda: Crustacea) with individuals losing up to three legs in a single moulting event [41]. Inversely, moulting in marine crustaceans can be triggered by limb autotomy [42,43] and it has been hypothesized that this occurs to prioritize limb regrowth [44]. However, it could also be a mechanism to proactively circumvent additional injury from moulting after regeneration has occurred. The close connection between autotomy and integral life patterns like intraspecific interactions and moulting highlights a need for further research into non-predatory entrapment drivers of autotomy in marine animals.

### (c) Injury reduction

The use of autotomy as a means of mitigating the cost of non-predatory injuries has also been noted but remains largely untested [19,25,45]. A recent analysis of two sacoglossan gastropods (Mollusca) revealed the use of autotomy as a unique mitigation technique when individuals were parasitized [46]. Rather than deal with the parasites via immune attack as is common in other taxa (e.g. sea cucumbers) [47], individuals of *Elysia marginata* and *E. atroviridis* autotomize their body from the head down which simultaneously removes the parasites before individuals regenerate a new posterior region [46].

It is hypothesized that while autotomy results in a wound, the additional adaptions immediately adjacent to breakage planes (i.e. adapted points of permanent weakness) facilitate a less costly recovery than other types of injury [25]. In a study of the cost of autotomy versus injury by ablation (i.e. manual removal of tissue/limbs) in the east Asian crab *Eriocheir sinensis* (Arthropoda: Crustacea), individuals recovering from ablation showed a higher rate of mortality than those having undergone autotomy of the same limb [48]. As injuries are a common by-products of non-predatory events or interactions (e.g. weather events, conspecific aggression), it is possible that selection for autotomy as a means of injury reduction could increase as anthropogenic influence drives large-scale environmental and biotic changes [49–51].

### (d) Foraging success

The increased expenditure of energy underlying the regrowth of tissue following autotomy generally needs to be supported through increased food intake. However, in many organisms, the loss of a limb through autotomy reduces foraging success by decreasing agility in prey capture (e.g. lizards; [52]) or food handling capacity (e.g. crabs; [53]), With potential cascading effects on the rest of the ecosystem. For example, a study of autotomy in the sea star *Heliaster helianthus* (Echinodermata: Asteroidea) suggests that arm loss could reduce its ability to consume its main prey, the periwinkle *Littorina littorea* and dogwhelk *Nucella lapillus*, shifting predator pressures and allowing population growth for the target prey [54].

To further explore the extent to which autotomy alters foraging patterns, researchers have developed flight initiation optimality models [55] that use the vulnerability of an organism to predict the amount of time it will spend in a refuge versus exposed to threats [56]. They can also assess the amount of time organisms will spend in the vicinity of a predator before fleeing, and be expanded to help understand the negative outcomes of autotomy on foraging [57]. According to these models, organisms that have recently undergone autotomy are more likely to seek refuge and forgo foraging opportunities compared to non-injured conspecifics [57–59], reducing overall pressure on prey populations and further supporting the trends described by Barrios *et al*. [54] in the sea star *H. helianthus*.

### (e) Reproductive success

Cases of voluntary limb loss that directly support successful mating have been widely documented across various taxa of marine and terrestrial invertebrates [37,60,61] and are often considered to be examples of true autotomy. While these behaviours meet three of the four defining criteria of autotomy (i.e. limb loss is voluntary, occurs at a predictable/consistent location and is supported by adaptive features), they do not occur in response to a threat or stress. Specifically, voluntary limb loss in those species was shown to increase fitness by ensuring successful copulation [3], in stark contrast with the usual tenet that one of the costs of autotomy is reduced reproductive fitness, whether that be temporary [54,62] or permanent [63]. The variable outcomes of limb loss in mating scenarios (Box 1) creates further ambiguity regarding the drivers of autotomy, warranting further research since reproductive success is such a strong driver of evolution.

Box 1 – **Autotomy and reproduction, a paradoxical relationship**

Smith [62] published the first experimental study showing that autotomy had a direct negative impact on reproductive success by examining mating patterns in the north atlantic crab *Callinectes sapidus* (Arthropoda: Crustacea). While males with missing legs were successful in pre-copulatory mating rituals, they demonstrated a reduced ability to guard potential mates from competitors, thereby reducing their overall reproductive success. This negative impact on mating success was only demonstrated in males, suggesting they may experience greater costs associated with voluntary limb loss than females. Whether the frequency of autotomic behaviour in *C. sapidus* is dependent on sex remains unclear, with some data suggesting females perform autotomy at higher rates [62,64] and other data finding no difference [65]. It is possible that the selective pressures described by smith [62] drives sexually asymmetric autotomy, with more females than males exhibiting voluntary limb loss [64,66]. Understanding how sex-specific costs of autotomy affect individual fitness, and by extension overall population dynamics, is crucial in characterizing the drivers behind this behaviour.

While autotomy is known to reduce reproductive fitness [54,67,68], a comparatively understudied number of species demonstrate increased reproductive fitness as a direct result of autotomy. For example, male octopi of the genus *Argonauta* (Mollusca: Cephalopoda) have evolved copulatory tentacles (hectocotyli) that autotomize and ‘swim’ towards the female to perform copulation. Through this adaptation, females are able to house and protect the autotomized tentacle containing male gametes until fertilization [69] and even hold onto multiple hectocotyli simultaneously [70], likely increasing fecundity and genetic diversity in offspring.

In populations with high spermatozoa competition (i.e. species where females mate with multiple males in a short time frame), autotomy supports reproductive success through the use of copulatory plugs [71]. Some species of nudibranchs in the genus *Glossodoris* (Mollusca: Gastropoda) autotomize their reproductive organs to act as a plug in the genital opening of their mate [60]. In these species, copulating males physically block others from successfully mating with the same female, thereby reducing competition. Alternatively, the nudibranch *Goniobranchus reticulatus* may use autotomizable appendages to remove spermatozoa from competitors (i.e. using backward facing spines on the penis to scrape out the vaginal canal) before copulation [27,60].

Finally, beyond reproductive success, there is some evidence that in the comatulid crinoid *Oxycomanthis japonicus*, autotomy can facilitate development in the juvenile stage [72]. In this species, arm autotomy is followed by bifurcation and regeneration, resulting in the development of an increasing numbers of arms [72,73].

## 3. Emerging and novel perspectives around autotomy

### (a) Incidence of injury

Since predator pressure is considered to be such a strong driver of autotomy [37,74], it can be expected that populations exposed to diminishing numbers of predators would also exhibit decreases in autotomic behaviour [49]. However, this trend is difficult to confirm, as most populations experience a multitude of other environmental and anthropogenic pressures that could cause wounding and/or stimulate autotomy [75]. It is possible that by consistently emphasizing predation as a prime driving force of autotomy, the more general pressure that injury (i.e. external tissue and/or skeletal damage that is not self-induced) may represent has been overlooked, i.e. it may be more accurate to look at overall incidences of injury as the evolutionary driver. Exploring the susceptibility of species to injury from a diversity of factors (e.g. predation, intraspecific competition, entrapment, weather, anthropogenic disturbances) may thus help shed light on the possible benefit of autotomy.

Lindsay [75] compiled a literature review on the frequency of injury in benthic marine invertebrates across several phyla to examine how the cost of injury and subsequent healing/regeneration impacts individuals and consequently influence how they interact with their environment. We modified and expanded the original dataset to make it representative of more marine ecosystems and allow us to examine it under the lens of autotomy. Specifically, this new dataset looks at the ‘frequency of injury’, measured as the average per cent of a population that demonstrates lost, healing/regenerating or scarred tissue in 116 species belonging to six phyla (Annelida, Arthropoda, Brachiopoda, Cnidaria, Echinodermata and Mollusca). For each species, the literature was reviewed to determine whether or not they were known to perform autotomy (electronic supplementary material, table S3). Additionally, as the term ‘frequency’ is often associated with a specified time frame, it was replaced with ‘incidence’ to clarify that the nature of the data is more general (average per cent of injured individuals per population) and was taken from variable sources. Using a chi-square test, and a Cramer’s V correlation, the relationship between the average incidence of injury and the proportion of species able to perform autotomy in each phylum was assessed.

**Figure 2.**
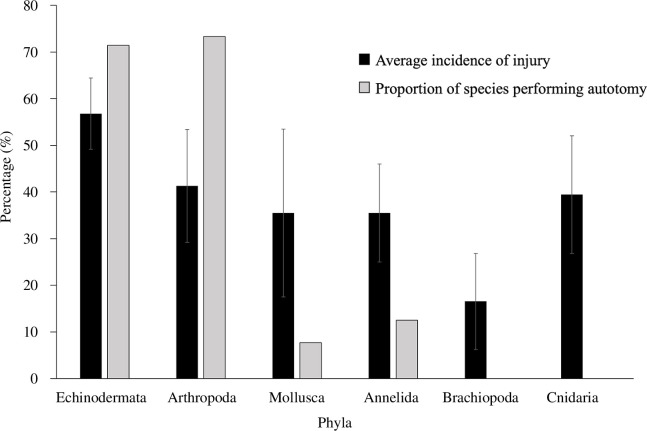
Average incidences of injury (percentage of populations demonstrating signs of injury through lost, damaged or scarred tissue; ± 95% confidence interval) in different phyla of benthic invertebrates (%) compared with the proportion of species able to perform autotomy (%) in the same six marine phyla. Modified dataset from [75] provided in electronic supplementary material, table S3.

Across all six phyla, species with a known ability to perform autotomy in some capacity experienced significantly higher incidences of injury (57% of individuals in a population; *p*<0.001) than those without this ability (37%; figure 2). The correlation analysis supported the strong relationship between autotomy and susceptibility to injury (Cramer’s V correlation value = 0.48). The significantly higher incidences of injury in species with autotomic abilities may point to a potential driver of this behaviour. It is also important to note that all species in the dataset have the ability to regenerate lost and damaged tissue to some extent, suggesting that regenerative ability is not the limiting factor. Taken together, these data support the hypothesis that, while autotomy is costly, it has the potential to allow organisms to occupy more physically challenging niches, possibly increasing the success of essential activities like foraging or reproduction.

Of the phyla included in the analysis, echinoderms (all classes except echinoids) and arthropods (specifically crustaceans) had both the highest incidences of injury and proportion of individuals able to perform autotomy (figure 2). In echinoderms, 57% of individuals in a population showed signs of injury, with 71% of representatives from classes Asteroidea, Crinoidea, Holothuroidea and Ophiuroidea having the capacity for autotomy (electronic supplementary material, table S3). Moreover, although arthropods showed lower average incidence of injury overall (41%), a similar proportion of their representatives demonstrated autotomy (73%). This further characterizes which taxa are more susceptible to injury and offers some insight into what factors (e.g. behavioural patterns or physiological characteristics) may apply selective pressure on autotomy. Additionally, the cnidarian species examined in this dataset showed incidences of injury that were comparable to arthropods while demonstrating no autotomic capacity. This suggests that while injury may be a strong driving force behind adaptive limb/tissue loss, it is likely not the only influencing factor and may need to act in combination with other selective forces to result in self-amputation (autotomy). Exploring the life-history strategies, anatomy and environments of taxa that demonstrate high levels of autotomy may help untangle these subtle potential evolutionary influences.

There are various factors that may alter the adaptive advantage of autotomy in arthropods and echinoderms. For instance, locomotive mode and speed, reproductive strategies and diet, all vary widely across these phyla and influence the levels of intraspecific aggression/competition and the rate of injury incurred as a result [76–78]. These two clades offer the opportunity to explore very different drivers of autotomy based on prominent differences in anatomy (skeletal and neural systems), place in trophic webs, means of locomotion and phylogenetic position. The remainder of this review explores under-researched drivers and outcomes of autotomy using echinoderms and arthropods as models where possible.

### (b) Anatomical specificities

Many organisms that perform autotomy do so at predetermined sites known as breakage planes, which are often proximal or integral to joints or vertebrae [4,10,29]. Species with anatomically jointed limbs offer various points of innate weakness [1,5] that could act as drivers for autotomy evolving along these planes. Assuming that limbs experience increased vulnerability based on weaknesses in articulation points, it would be intuitive for limbs prone to injury to experience selective pressures favouring the emergence of autotomy [45].

Echinoderms and arthropods provide excellent case studies for the evolution of these types of breakage planes. For example, brittle stars (Echinodermata: Ophiuroidea) submitted to tactile stimuli can undergo autotomy at articulation points occurring along the length of the arm (figure 2; [79]). Specifically, autotomy in this class occurs along so-called ‘intervertebral’ breakage planes, where splitting is supported by permanent epidermal weakness and nervously destabilized tendons and mutable connective tissue (MCT), which is also characteristic of all echinoderm autotomy (figure 2; [10,80,81]). The fact that limb loss can be induced at any of these points along the arm and that this class demonstrates rapid regeneration both help maximize efficacy of autotomy as a behaviour in brittle stars (figure 2; [30,79,82]).

**Figure 3.**
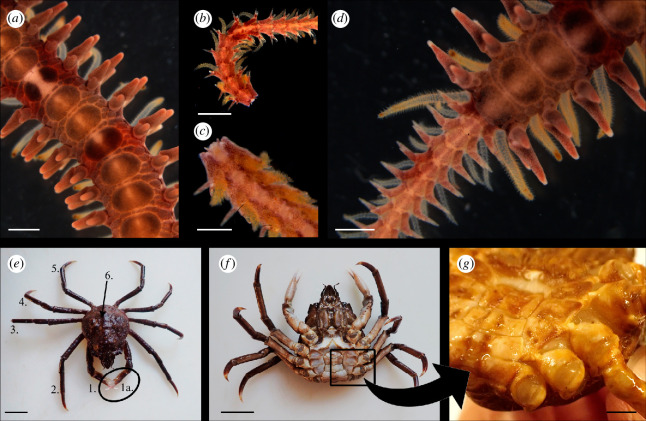
(*a–d*) Breakage planes in the arm of the brittle star, *Ophiopholis aculeata*. (*a*) Full, unautotomized arm (scale bar = 0.5 mm), (*b*,*c*) autotomized arm, (*b*) dorsal surface (scale bar = 0.5 mm), (*c*) ventral surface (scale bar = 0.25 mm), (*d*) regenerating arm (scale bar = 0.5 mm). (*e–g*) Breakage planes in the legs of the toad crab (*Hyas coarctatus*) (*e*) dorsal view with numbers 1–5 indicating legs (pereopods; scale bars = 30 mm), 1a = cheliped, 2–5 = walking legs, 6 = carapace. (*f*) Ventral view (scale bars = 30 mm), arrow indicates (*g*) is a close-up of the section outlined in black box. (*g*) Breakage planes with anteriorly lost pereopods (scale bar = 12 mm).

While autotomy in echinoderms is supported largely by destabilization of MCT, thereby preserving major muscle groups [83,84], autotomy in decapod crustaceans like crabs relies primarily on muscular force [85]. In the loss of pereopods, the posterior levator contracts, forcing rotation of the small tendon, in turn causing the anterior levator to snap at a preformed breakage plane (figure 3; [5,41,86,87]). This occurs at the narrowest, and consequently weakest point in the pereopods [45]. Most echinoderms and arthropods demonstrate autotomy within or proximal to an anatomical articulation, suggesting that the tissues, muscles and skeletal elements present in these locations may act as drivers for emergence of autotomy within a species.

Contrary to the autotomy seen in taxa with anatomical articulation points, there are a few cases where voluntary limb loss can occur in non-segmented organisms [88]. The best-documented case of this is evisceration, i.e. the voluntary expulsion of internal organs, in sea cucumbers (Echinodermata: Holothuroidea; [89]). This autotomic behaviour occurs for various reasons, including to escape from a predator [22] or defend against parasites [90], and may also be due to seasonal challenges (e.g. aestivation; [91,92]). How this voluntary evisceration is demonstrated can vary across taxonomic orders of holothuroids. For example, it occurs anteriorly in Dendrochirotida via the discarding of the aquapharyngeal bulb and integument of the oral region (e.g. *Sclerodactyla briareus;* [93]) and posteriorly through the anus in Holothuriida (e.g. *Holothuria parva;* [94]). While there is no anatomical articulation point to visually indicate exactly where the tissues will break, autotomy still occurs at predictable locations (e.g. across a breakage plane) to allow for the loss of specific organs. For example, holothuriids always eviscerate the digestive tract, haemal vessels and the respiratory tree [95]. Evisceration is mediated by the irreversible destabilization of MCT (the same mechanism facilitating all echinoderm autotomy, see above discussion on brittle stars [10]). In addition to the voluntary breakdown of tissue, autotomy of viscera is supported by muscular contractions [88].

A lesser known, albeit equally interesting form of autotomy, involves transversal scission leading to the rapid loss of a posterior portion of the body, followed by regeneration of the anterior end, in members of the holothuroid order Apodida [75,96,97]. Transversal scission differs from transversal fission, which is a mode of asexual reproduction that occurs over a longer period and results in both halves of the holothuroid being regenerated (i.e. multiplication). Autotomic scission has been documented in only a few species, including *Chiridota laevis* [96], *Leptosynapta clarki* [98], *L. inhaerens* [99], *Polycheira fusca* [86,97] and *Synapta maculata* [100]. In all investigated cases, it appears to be triggered by stressful physical contact (e.g. predator attack) leading researchers to believe it is a form of defence. Like evisceration discussed above, voluntary transversal scission is an interesting autotomic peculiarity, as there is no defined breakage; the body can be severed anywhere between the point of stimulus and the anterior end [97]. This autotomic split presumably still relies on MCT (although this has yet to be studied) to destabilize tissue at the site of scission and uses muscular contraction of the body to both separate the halves and constrict/close the wound site [96,97]. The appearance of autotomy in holothuroids in the absence of a visually delineated or articulated breakage plane provides opportunities to expand our understanding of the purpose and evolutionary drivers behind the phenomenon.

### (c) Cellular activity

Various possible adaptations support the efficiency of autotomy by helping to alleviate its cost, including the ability to heal rapidly. Characterizing cellular healing responses post-autotomy is thus pivotal. Cells known as coelomocytes are foundational to invertebrate innate immunity and are thought to be key players in supporting wound healing and regeneration [101–103].

Beyond providing a marker of stress [104], coelomocytes could be used to expand our understanding of the cellular response triggered by autotomy. One promising study [48] on the crab *E. sinensis* (Arthropoda: Crustacea) noted that, following autotomy, immune cells rapidly increased, both from tissue recruitment/migration and due to cell proliferation. The cellular spike was much shorter (i.e. coelomocytes rose in number for a shorter period of time) in cases of autotomy (1 h) than in cases where limbs were ablated (2–3 h; [48]) , indicating that a lower stress response was invoked by voluntary than by forceful limb loss. However, in another crab (*Carcinus aestuarii*) that cannot perform autotomy, limb ablation caused an initial spike in immune cells through recruitment and migration, similar to *E. sinensis*, but cell proliferation did not show an increase until 7 days later [105]. Based on these findings, Yang *et al*. [48] proposed that capacity for cell proliferation varies greatly between species. The results of these two studies suggest that the capacity for a species to support healing and regeneration through rapid cellular proliferation may act as a physiological underpinning of autotomy.

### (d) Environmental pressures

A foundational principle of autotomy is its nervous mediation [9,106] which, over the last 150 years, has been described using many terms (e.g. intelligent, reflexive, conscious, voluntary) each of which denotes varying levels of organismal control [15,33,107,108]. The disparate terminology used to describe the moderating mechanism of autotomy has possibly introduced some confusion regarding its plasticity. For example, it has been suggested that in order for a species to exhibit true autotomy, limb loss cannot be influenced by season or development [3]; however, there is documented evidence suggesting that this is not always the case. Fleming & Bateman [45] noted that organisms held in captivity or exhibiting poor health were less likely to perform autotomy. Additionally, crabs that had recently undergone loss of one or more legs were noted to require elevated stress levels to further autotomize [5]. These seemingly simple observations point to a largely understudied idea, namely that environmental changes can drive autotomic plasticity.

There are a handful of examples where individuals of a species alter threshold requirements for autotomy, whether this be following tactile or environmental stimuli [109–111]. However, two areas that require more research are shifts in environmental conditions due to climate change (e.g. ocean warming, and changes in salinity, oxygen and nutrient levels) and increased anthropogenic pollution. Few experimental studies have explored these angles at the individual or population levels. As climate change is widely anticipated to impact interspecies interactions [112], it is important to understand how autotomic behaviours may be affected and, subsequently, how this will alter the way organisms interact with and support their ecosystems. Changing autotomic behaviours in response to climate-related environmental shifts have been noted only a handful of times, primarily in echinoderms and arthropods.

A study by Rome *et al.* [113] examined the physiological metrics of stress in the crab *Callinectes sapidus* following fluctuations in temperature and salinity, and found that young individuals undergoing periods of stress could autotomize non-vital limbs and re-allocate this maintenance energy to vital organs. However, all autotomizing individuals in the experiment eventually died from prolonged exposure to harsh conditions, indicating that autotomy cannot help individuals acclimate to new salinities/temperatures and that limb loss is possibly only a short-term response to preserve energy until conditions stabilize. A study of the brittle star *Ophiophragmus filograneus* produced similar results [114], whereby exposure to both extreme high (39 psu) and extreme low (8 psu) salinities produced increased incidences of autotomy followed by eventual death.

While changes in temperature and salinity seem to consistently increase rates of autotomy, exposure to pollution produced more variable responses. When exposed to organophosphate pesticides, the crab *Callinectes sapidus* demonstrated a greater affinity for autotomy [115]. This trend was seen in another crab, *Chionoecetes bairdi*, exposed to crude oil, where recently moulted individuals showed greater susceptibility to voluntary limb loss than those with harder carapaces [116]. Those that died also demonstrated the highest number of autotomized limbs. Inversely, individuals of a third species of crab, *Hemigrapsus sanguineus*, that were exposed to high levels of nitric oxide showed delayed or absent autotomic behaviour [117]. The varied autotomic responses to abruptly changing conditions exemplified here demonstrate how nuanced the drivers of this behaviour are. Without more experimental research in this area, it remains difficult to predict what the long-term ecological outcomes will be. Based on this handful of cases, it is plausible that the impacts of anthropogenic activities, such as accelerated climate change and pollution, on complex responses like autotomy will have deep and long-lasting repercussions on marine ecosystems.

## 4. Potential applications of autotomy

The costs–benefits of autotomy are generally explored within the context of organismal fitness, including the ability to heal, forage, reproduce and defend against predators. While many organisms that perform autotomy experience increased vulnerability and reduced fitness for at least a period of time (if not permanently) [44,118,119], some species have been found to experience relatively minor effects from autotomizing a limb [120]. In turn, these apparently milder outcomes of autotomy relative to traumatic injury have given rise to surprising practical applications.

The claw-only harvesting of decapod crustaceans [121] was implemented to prevent the removal of mature organisms from a population, under the assumption that they can regenerate and be fished multiple times [122]. However, various studies have reported high rates of disease and mortality in forcibly de-clawed crabs (i.e. in crabs whose claws have been amputated; [121–123]), suggesting that they may not be re-integrating into their ecosystems as seamlessly as originally thought. To mitigate this, industry experts in various fisheries (including the stone crab, brown crab, fiddler crab and Jonah crab fisheries) have pioneered the use of inducing claw autotomy as an alternative protocol for de-clawing via amputation [124,125]. Initial investigation from researchers at the Cape Eleuthera Institute^1^ demonstrated that autotomized crabs have higher survival rates and are able to participate in foraging, movement and mating in a relatively normal way [66,120,126]. However, research from the stone crab industry indicates that current de-clawing practices do not consistently result in induced autotomy, meaning forced de-clawing still occurs at a high rate, suggesting that further research in this area is needed to increase the efficacy of fishing practices [123,127,128].

Another emerging use for autotomy in the commercial harvest of crustaceans relates to soft-shell crabs, which does not refer to a species but rather to the moult stage (ecdysis) where crabs have recently shed their hard exoskeleton revealing a new, soft one beneath [129]. Within these fisheries, autotomy is a potentially useful protocol as it has been shown to trigger moulting, which decreases the intermoult period [78], reduces intraspecific aggression [120] and makes the harvesting of soft-shell crabs more predictable. Fisheries use autotomy (induced through puncturing tissue proximal to the breakage plane or other tactile stress) to trigger moulting, which is the preferred harvesting phase for this industry [130,131]. Assessments of this new method are currently not in agreement. Two studies showed that the accumulated effects of repetitive, forced moulting eventually resulted in slower growth and generally smaller body size [131,132] while another found that it made no difference [131]. As the value of soft-shell crabs can depend heavily on size, decreasing body size in commercially harvested populations would be counterproductive. Due to the ambiguous results, more research is needed to determine if inducing rapid molt cycles through autotomy is economically and ecologically sustainable for these fisheries.

Similar to the crustacean examples, some holothuroid fisheries have begun relying on the regenerative potential of their commercial species in an effort to avoid removing mature individuals from the environment [133,134]. Holothuroids, or sea cucumbers, are one of the world’s most lucrative sea food. While the industry is largely focused on the body wall marketed as beche-de-mer, there is substantial demand for the internal organs (viscera) which are sold as a fermented food delicacy (specifically the intestines and gonad; [135]) and a resource for the pharmaceutical industry [136].

While there are no sea cucumber fisheries currently using autotomy as a means of harvesting, several studies have examined the efficacy of regeneration in current fisheries [133,134], and thus provide a foundation for exploring autotomy as an alternative. Currently, fisheries that harvest viscera commercially (primarily in the Pacific Islands) do so manually [137], i.e. the body wall is incised to remove the internal organs and the individual is returned to its environment [133]. This method is practised primarily on posteriorly eviscerating sea cucumbers (e.g. *Stichopus horrens*). Sea cucumbers that have autotomized their viscera can rapidly regenerate their internal organs, some in as little as two weeks [138–141]. While studies show that sea cucumbers that have their viscera manually removed re-grow their organs at a similar rate than those that eviscerate via autotomy [133], other metrics of wellbeing (e.g. weight and immune responses) suggest that manual removal of organs is more detrimental [133]. For example, a study by Zang *et al*. [89] demonstrated that *Apostichopus japonicus* lost about 13% of its body weight while regenerating autotomized internal viscera compared to a study by Charan-Dixon *et al*. [133] who demonstrated that individuals of *S. horrens* that were manually eviscerated lost about 26% of their body weight throughout regeneration. There is also some dispute regarding survival following manual evisceration, with studies reporting variable survival rates of 57–98% [134,142]. However, in studies where sea cucumbers were induced to autotomize the viscera, the survival rates were consistently greater than 95% [94,143,144]. These findings suggest that inducing the autotomy of internal organs as a means of collection, as opposed to opening the body wall to manually removal viscera, may support the design of more sustainable practices going forward.

## 5. Conclusions

Drivers of autotomy can exist at many organizational levels, including cells (e.g. coelomocytes), organisms (e.g. skeletal/muscular weakness), populations (e.g. intra/interspecific aggression) and whole ecosystems (e.g. anthropogenic changes). The outcomes of each of these levels have the potential to shift the adaptive advantages of autotomy and thereby create a highly complicated web of species-specific responses to changing conditions. While previous research on voluntary limb loss in terrestrial reptiles (e.g. tail loss) has been crucial in illustrating key aspects of the relationship between organisms, autotomy and their surrounding environment, it fails to adequately capture the drivers and outcomes of autotomy in marine taxa. Marked differences in environmental pressures and anatomical organization emphasize the need for taxonomically diverse assessments, including more marine model organisms, in this field of research.

Beyond the fundamental outlook, applied aspects are emerging. The practical impact of designing subsistence fisheries is becoming increasingly significant in providing food and financial security to communities across the world. The minimalist ideology of harvesting only what is necessary speaks to the general principles of environmental sustainability, and the promising results of emerging research around autotomy support this. However, more experimental work, primarily *in situ* research on the community impacts of autotomy, needs to be conducted before the use of autotomy as a method for de-clawing commercial crab species and eviscerating sea cucumbers can be implemented as a standard protocol. Furthermore, the long-term effects of consistently induced autotomy need to be investigated at population levels. Ongoing research into the commercial applications of autotomy will hopefully help design more sustainable fisheries.

This review has highlighted the potential of echinoderms and arthropods (primarily crustaceans), to act as key model phyla in supporting future work in this discipline. Beyond the many anatomical and ecological benefits listed previously, the prolific occurrence of both groups globally would allow for research addressing temporal vulnerabilities. Moreover, the placement of echinoderms as basal deuterostomes makes them a logical intermediate model organism to expand autotomy research beyond vertebrates.

## Data Availability

The supporting data is available as part of the electronic supplementary material [145].

## Footnotes

^1^
The campaign, ‘Don’t clip the biter, stick it’ is currently being pioneered by the Cape Eleuthera Institute in conjunction with this research.
